# Immersive Virtual Reality for Enabling Patient Experience and Enrollment in Oncology Clinical Trials: A Feasibility Study

**DOI:** 10.3390/cancers17071148

**Published:** 2025-03-29

**Authors:** Frank Tsai, Landon Gray, Amy Mirabella, Margaux Steinbach, Jacqueline M. Garrick, Nadine J. Barrett, Nelson Chao, Frederic Zenhausern

**Affiliations:** 1 HonorHealth Research Institute, Scottsdale, AZ 85258, USA; amirabella@honorhealth.com (A.M.); msteinbach@honorhealth.com (M.S.); jagarrick@honorhealth.com (J.M.G.); 2 Center for Applied Nano Bioscience and Medicine, College of Medicine, University of Arizona, Phoenix, AZ 85004, USA; 3 Atrium Health Wake Forest Comprehensive Cancer Center, Wake Forest School of Medicine, Winston-Salem, NC 27157, USA; njbarret@wakehealth.edu; 4 Maya Angelo Center for Health Equity, Wake Forest School of Medicine, Winston-Salem, NC 27101, USA; 5 Department of Medicine, Duke University School of Medicine, Durham, NC 27710, USA; nelson.chao@duke.edu; 6 Department of Basic Medical Sciences, College of Medicine, University of Arizona, Phoenix, AZ 85004, USA

**Keywords:** digital health, immersive technologies, virtual and extended reality, VR/XR, oncology, cancer, informed consent, clinical trials, healthcare

## Abstract

**Background/Objectives**: Informed consent is a crucial part of the clinical trial enrollment process in which patients are asked to understand and provide approval for medical interventions. Consent forms can be complex and hinder patient comprehension, highlighting the need for novel tools to improve the patient enrollment experience. This feasibility study aimed to develop an immersive technology to enroll human subjects in oncology clinical trials and provide 3D avatar-based informed consent in a virtual reality (VR) environment. **Methods**: Clinical feasibility and the effects of head-mounted VR devices on motion sickness and educational quality were evaluated in adult oncology patients enrolled in an intravenous (IV) port placement intervention study. Participants received before- and after-questionnaires to measure their understanding of the information received in VR. A follow-up questionnaire was given four weeks post-consent to measure knowledge retention. **Results**: Clinical staff reported that VR technology was manageable to use. Among 16 adult participants, all reported that VR was well tolerated with no motion sickness. The mean pre-intervention knowledge score was 64.6%, with an immediate post-intervention knowledge score of 97.9%. A mean knowledge score of 93.3% four-weeks post-consent was observed among 10/16 participants who completed a follow-up questionnaire. **Conclusions**: These findings support that VR is well tolerated and effective at delivering information during the informed consent process for oncology clinical trials. Key limitations include the small sample size and single clinical population. Further trials are warranted to compare efficacy over traditional consenting mechanisms and include more diverse clinical populations among a wider participant pool.

## Simple Summary

Informed consent, wherein a patient receives information on a specific medical intervention and provides agreement to proceed, is a vital step in the clinical trial enrollment process that can be taken to ensure that patients understand the research study and their rights as trial participants. Informed consent documents can be difficult to understand for many reasons, resulting in patients being confused about the trial in which they are participating. By simplifying the informed consent process, technology may be able to improve the patient experience during clinical trial enrollment. Our feasibility study aimed to assess the feasibility of using an immersive virtual reality experience to improve the patient enrollment process in oncology clinical trials. Among 16 adult oncology patients enrolled in an intravenous port placement study, we found the virtual reality experience was well tolerated, caused minimal motion sickness, and led to high information retention with recall testing. These results support further assessment of this immersive virtual reality experience for use in oncology clinical trial enrollment.

## 1. Introduction

Clinical trials are the cornerstone of advancing therapeutics and precision medicine. However, adequate accrual of participants with characteristics that represent eligible patients with a particular disease condition or high-risk factor remains a daunting challenge. The enrollment process includes informed consent, which is a process of communication between a physician and a patient that leads to the patient’s authorization to proceed with a specific medical intervention [1]. The patient’s consent for the medical procedure must occur with free will, independent of the physician’s opinion, and is considered valid only once the management alternatives, the expected benefits, and the potential risks have been explained and comprehended [2,3].

To ensure that proper informed consent is achieved before a procedure, a physician must ensure that (1) a clear description of the proposed intervention is provided, (2) the decision-making authority rests entirely with the patient, (3) alternative interventions are discussed, (4) all associated risks of the proposed intervention are discussed, and (5) the patient provides a decision independently [4]. Studies have shown that a lack of understanding or misunderstanding of surgical procedures can increase preoperative anxiety, which can mean that patients require more sedation and anesthesia, which can lengthen recovery times and increase the need for medical intervention postoperatively [5,6]. The informed consent process must emphasize educating patients on potential complications before a surgical procedure and ensure that a patient’s knowledge is not restricted by poor health literacy, language barriers, or anxiety, which could lead to a lack of patient understanding [4,7]. Inaccurate or poorly documented informed consent about a clinical procedure, combined with the patient’s expectations of treatment outcomes, could leave a patient misinformed and increase the risk of a legal conflict [8].

Another important consideration when drafting informed consent documents is the reading comprehension level of the document. Physicians have an exceptionally high reading level compared to the general public owing to their rigorous education and professional application of reading at the highest scientific level. While physicians are expected to write informed consent documents with wide readability in mind, the readability of a document can be difficult to judge. Both physicians and the institutional review boards tasked with review of clinical trial documents can overestimate the reading level of the average person and produce documents that are too complex [9]. Indeed, while most healthcare materials are written at the 10th-grade reading level, most adults only read at the 8th-grade level, with nearly 20% of the population reading at or below the 5th-grade level [10]. Those who read at lower levels may struggle to complete necessary forms proficiently while being ashamed to ask for assistance, leave with unanswered questions, or sign documents that they do not fully understand [10]. Despite efforts to eliminate this gray area between care providers and patients, satisfactory comprehension can be difficult to achieve owing to the use of medical jargon during communication with patients, the psychological status of the patient, or the use of traditional paper-based consent forms that may limit clinicians’ ability to pinpoint details specific to their patient’s case, as they often limit the dialogue to the mere reading of the document [2,11].

With the constantly expanding societal use of computers for medical information, digital multimedia may bridge the literacy gaps between physicians and patients [12,13]. Although the same concerns exist regarding the amount of information, ability to comprehend, and formatting apply regardless of the mode of information delivery, digital multimedia offers several important advantages over traditional paper consent forms, such as promoting active participation, the ability to tailor information to specific patients, and the potential to incorporate interactive in-line exercises that can establish an understanding of information at the time that decisions are made [14]. A specific application of this potential improvement using digital multimedia technology is the prospect of using extended reality (XR), which includes virtual reality (VR), augmented reality (AR), and mixed reality (MR), which all have the potential to enhance consent processes by providing an immersive, interactive, and multimodal sensory experience that aids in patient education [4].

A specific application of improving clinical trial enrollment via XR technology platforms involves enhancing clinical trial enrollment in underserved populations by promoting health literacy and access to clinical trials relevant to a patient’s personal needs, socioeconomic environment, cultural preferences, and other societal values. Another profound impact of poor health literacy is that it limits access to oncology clinical trials, particularly among racial and ethnic minority populations, people who live in rural communities, those with low socioeconomic status, and other underserved populations [15,16]. Oncology clinical trials are necessary to determine the efficacy and potential adverse effects of novel anticancer therapies, with the goal of improving cancer health outcomes and advancing health equity. However, only 2–3% of adult cancer patients participate in clinical trials, and underrepresented racial and ethnic population participation is especially limited [17,18]. Access to clinical trials relies mainly on the patient’s physician making such a recommendation, which highlights significant impediments to enrollment, as physicians may have implicit biases, lack available information about trials, or lack staffing resources to spend the time finding and determining the most appropriate trial for an individual patient.

In modern surgery, both AR and VR are used in preoperative planning and intraoperative navigation, which has increased the quality and safety of surgical procedures [19]. Because of these successes over the past decade, the application of VR technology has expanded to explore the effects of VR simulation on physical rehabilitation, pain management, surgical training, anatomical education, and the treatment of psychiatric disorders [20]. By integrating various technologies, including head-mounted displays (HMDs) with head-tracking systems, headphones for sound/music and noise-cancelation, and manipulation/navigation devices, VR technology creates a multisensory, three-dimensional (3D) environment that allows users to become fully immersed in a simulated world [21]. A visual display using virtual features of the patient’s medical condition (e.g., a 3D-generated model from medical imaging of an individualized tumor), treatment options, risks, intervention methods (e.g., surgery, radiotherapy), and benefits of procedures have been proposed to enhance and relieve uneasy feelings before a clinical intervention [22]. While VR has demonstrated exciting possibilities in the area of medical education, some limitations such as cost, motion sickness in users, and the lack of standardization within the VR industry must be considered when developing VR for clinical use [23,24,25].

The use of VR for informed consent within oncology clinical trials remains minimally explored and it is unclear how effective this technology may be in this space. We hypothesize that VR can be used as a powerful medical education tool during the informed consent process for patients to enable the learning of complex medical interventions and improve the enrollment experience in oncology clinical trials for both patients and clinical staff. To explore this hypothesis and address the current knowledge gap, the primary objective of this feasibility study was to assess the feasibility of using VR digital technology in a clinical setting to improve the patient experience during the informed consent process for an intravenous (IV) port placement procedure, which is a commonly performed procedure in oncology trials. Additionally, evaluation of procedure knowledge received during the VR experience is reported to determine the educational benefit of the technology.

## 2. Materials and Methods

### 2.1. Study Overview

This was a single-center, multiple-provider study with patients who had received a cancer diagnosis. Some patients were actively in need of an IV port for infusion therapies, while others were not. Patients were recruited from the HonorHealth Virginia G. Piper Cancer Center (Phoenix Metropolitan Area, Arizona, USA). The HonorHealth Institutional Review Board approved the study (IRB protocol # 1467535).

### 2.2. Participants

The criteria for inclusion were the age of 18 years or older, were English-speaking, had the ability to provide informed consent, and had an oncological diagnosis. Patients were excluded if they were non-English-speaking or had brain metastasis. Recruitment was initially designed from January 2020 to April 2022.

Initially, the patient’s physician(s) having requested IV port placement was an inclusion criterion and prior port placement was an exclusion criterion. After study activation, it was determined that this criterion biased the recruitment population and put an undue challenge on these patients, as most patients in need of a port with no prior port placement were newly diagnosed cancer patients who had a significant burden of frequent medical visits for staging workup, including radiologic studies and biopsies. Study activation also coincided with the advent of the COVID-19 pandemic, during which in-person clinic visits were suspended. Owing to these barriers, the protocol was amended to include all oncology patients irrespective of prior port placement or port need and whether they were newly diagnosed, in active treatment, or under surveillance. The enrollment period was extended to April 2022 due to limited in-person activity during the COVID-19 pandemic.

### 2.3. Study Process

#### 2.3.1. Pre-Screening

Upon being referred for the feasibility study by their treating oncologists, patients were contacted by phone by the research nurses, who provided an overview of the study. If agreeable, patients were pre-screened by the research nurse with the intervention visit scheduled at an offsite location at the HonorHealth Research Institute, which is located a short walk across from the oncology clinic. The intervention visits were all before the actual port placement date.

#### 2.3.2. Intervention Visits

During the intervention visit, the research nurse informed the participants of the study details and answered additional questions from the patients. After this, patients were considered consented for the study. Following the study informed consent process, patient demographic information was collected, and two questionnaires were administered: (1) a three-question questionnaire to assess each patient’s knowledge about IV ports and (2) the eHEALS questionnaire to assess each patient’s combined knowledge, comfort, and perceived skills in evaluating electronic health information competence.

The three-question knowledge questionnaire asked the following: (1) Where in your body will the port be placed? (2) List two symptoms of problems that could occur following the placement of the port that would cause you to call the clinic right way. The content has only 1 complication. (3) Please identify two things the patient should not do while the medical glue is still on the incision. All knowledge assessment questions were free-response and correctness was scored at the discretion of a physician. Assessment questions were designed at the 6th-grade reading level, utilizing a universal precaution approach to health literacy (AHRQ/DHHS). The eHEALS questionnaire is an eight-question measure of electronic health information literacy developed to measure individuals’ combined knowledge, comfort, and perceived skills in finding, evaluating, and applying electronic health information to health problems [26]. Low electronic health information literacy is defined as a score <24, with moderate-to-high electronic health information literacy defined as a score ≥24.

**Figure 1:**
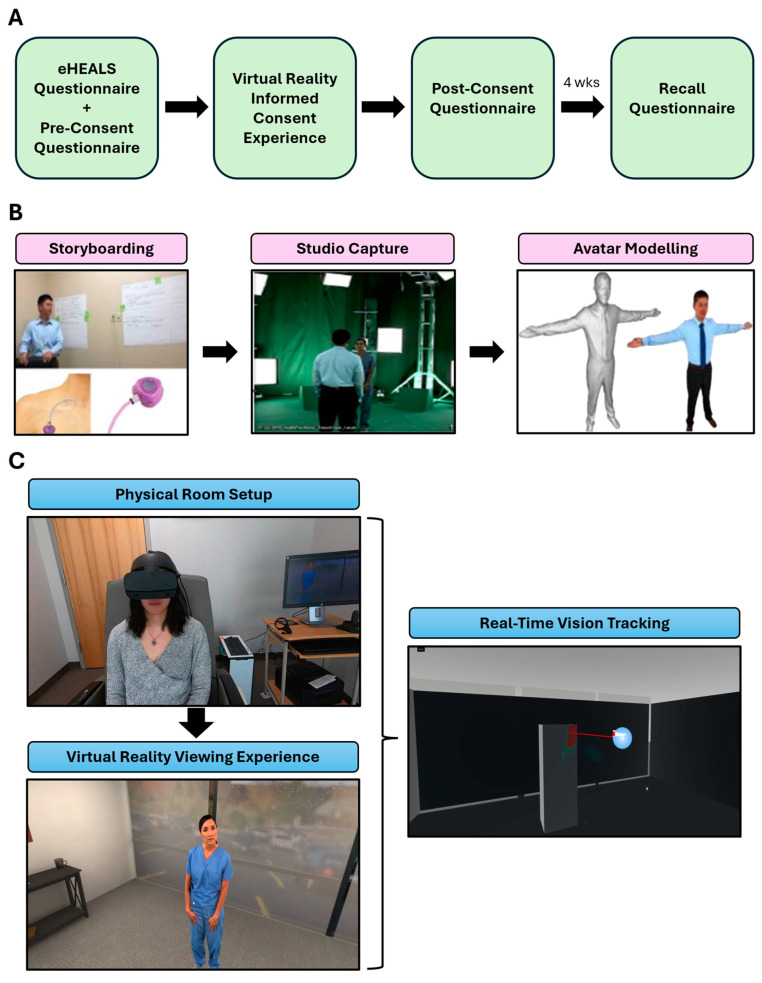
(**A**) Summary flowchart of the study; (**B**) overview of VR technology creation. The process included the use of storyboarding to generate scripts for IV port information, 3D studio capture of avatars for use in a VR environment, and the use of volumetric computing to generate VR avatar models and environments. (**C**) Overview of the patient VR experience. A view of the physical room the participant is in, the visual field of the participant when wearing the VR headset, and an example of real-time eye tracking capabilities of participants.

The VR education and informed consent intervention consisted of a one-minute, forty-second (00:01:40) experience in a VR environment simulating a comfortable office setting with the patient sitting in a chair. During the digital immersive session, the patient sees a female avatar that speaks to the patient and provides information on port placement and management. The avatar can make eye contact and turn her head to maintain eye contact with the patient-user (Figure 1C). The virtual reality content was provided by Microsoft and developed and produced at Microsoft Mixed Reality Capture Studios (San Francisco, CA, USA). The production involved the filming of two of the clinical investigators, who played the roles of patient and clinician. The avatars of the actors were created in the VR environment via spatial programming [27], where the viewer may experience the content from the perspective of the patient, clinician, or third person (Figure 1B; Supplemental Video S1). Patient engagement was assessed via a validated instrument prior to the intervention, immediately following the educational intervention, and four weeks after the educational intervention. When available, all other self-reported assessments were conducted at this time (Figure 1A).

The VR experience was used in place of traditional written documentation for informed consent. The VR intervention included a minimum of three sessions of one-minute, forty-five-second (00:01:45) VR exposure, with the final session anticipated to reach a criterion of 95% comprehension improvement from the baseline assessment between the clinician and participant. The virtual reality hardware included an HTC Vive VR headset (HTC Corporation, Taoyuan, Taiwan) using software from the Microsoft library of metaverse tools used at Microsoft Studios (San Francisco, CA, USA).

The team of clinical investigators at the HonorHealth Research Institute (HRI) designed the content for this study starting from a script design using storytelling board techniques (Figure 1B). The primary objective of the feasibility study was to demonstrate that VR technology can be successfully deployed in a clinical research environment. The additional primary objective was to assess the potential adverse effects of VR technology (e.g., motion sickness) on cancer patients. The secondary objectives included immediate and long-term knowledge retention by patients who received informed consent information through a VR environment. The primary endpoint was the development of a comprehensive patient profile that could assess the experience of participating in the VR platform. A combination of data sources was synthesized (patient medical records, video/audio recordings, transcripts from semi-structured interviews, eHEALS scores, tolerability indices (such as nausea), and patient demographics) to provide a comprehensive understanding of the experience. The knowledge questionnaire results were used as the secondary endpoint to assess the learning metrics of patients engaging in the VR platform. The outcome measures included changes in pre- and post-test scores on the investigator-developed port placement and care questions from baseline to immediately following the VR experience (termed “intervention”) and after 4 weeks.

### 2.4. Statistical Analysis

To assess knowledge retention and the effectiveness of the intervention, a three-question questionnaire was administered pre-intervention, immediately post-intervention, and 4 weeks post-intervention. The mean percentage of correct answers for each questionnaire timepoint were compared using a Restricted Maximum Likelihood (REML) mixed-effects model with Tukey’s multiple comparisons post hoc test. This model is particularly suitable for analyzing data with repeated measures, such as in this study where the same individuals were assessed at multiple timepoints. All statistical analyses were performed using GraphPad Prism software version 10.4.0 (San Diego, CA, USA).

## 3. Results

### 3.1. Participant Summary

A total of 16 patients (*n* = 16) with cancer diagnoses were enrolled in the study. The mean age was 65 years and ranged from 50 to 88 years. All patients had an Eastern Cooperative Oncology Group (ECOG) Performance Status Scale score of 0 or 1.

### 3.2. Clinical Implementation of VR Technology and Patient Tolerance

The VR technology was deployed successfully in the clinic, requiring minimal expertise from the clinical staff. All patients tolerated the treatment well without use-limiting adverse effects. One patient reported feeling startled by the avatar but was able to continue the consent process. No patients experienced vertigo or nausea. All patients tolerated the hardware, including one patient with nasopharynx cancer who had recently completed chemoradiation. Technical challenges included patients reporting that the audio volume was low and sometimes difficult to hear.

### 3.3. Electronic Health Literacy Scale (eHEALS) Scores

**Figure 2:**
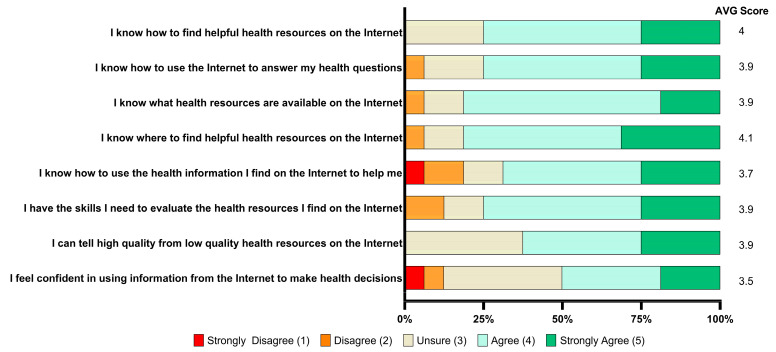
Results of the Electronic Health Literacy Scale (eHEALS) Questionnaire. The questionnaire scores range from 8 to 40, with low electronic health information literacy defined as a score < 24 and moderate-to-high electronic health information literacy defined as a score ≥ 24.

Patients were assessed for electronic health information literacy via the 8-question eHEALS questionnaire. The average scores on a question-by-question basis ranged from 3.5 to 4, with a total average score of 30.9 (95% CI [28.2,33.6]), indicating moderate-to-high electronic health information literacy in this patient population (Figure 2).

### 3.4. Pre-Intervention, Post-Intervention, and Recall Intervention Knowledge Scores

**Figure 3:**
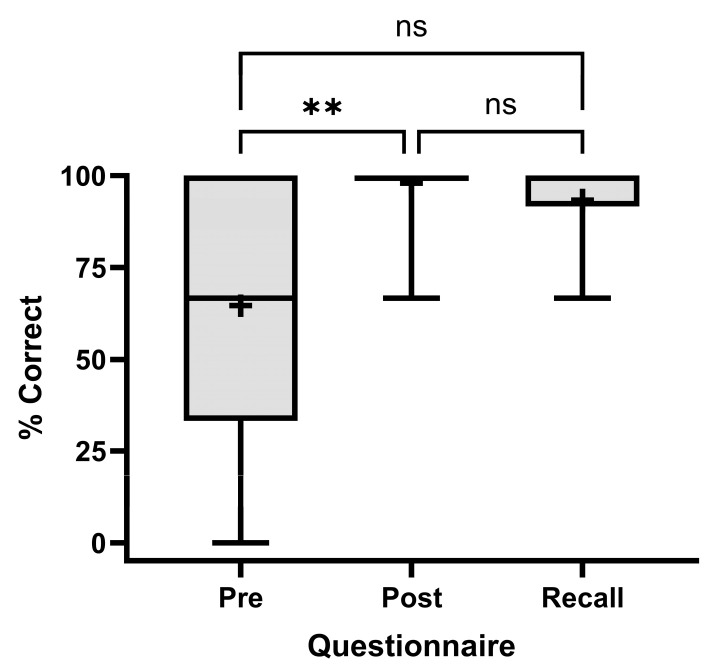
Percentage of correct answers from a three-question questionnaire assessing IV port placement knowledge. The questionnaire was administered pre-intervention (“Pre”, *n* = 16), immediately post-intervention (“Post”, *n* = 16), and four weeks post-intervention (“Recall”, *n* = 10). ** *p* < 0.01, ns *p* > 0.05, using a REML mixed model with Tukey’s multiple comparisons test. Boxes extend from the 25th to 75th percentile, with the middle line representing the median; the mean is represented by + sign; extending lines denote minimum and maximum values.

Using a three-question questionnaire assessing knowledge about IV port placement, the mean pre-intervention knowledge score was 64.6% (95% CI [45.7, 83.5]). The mean immediate post-intervention knowledge score was 97.9% (95% CI [93.5, 100.0]), with an absolute improvement of 33.3% over the pre-intervention score. Of 16 participants, 10/16 (63%) completed the four-week post-intervention recall questionnaire, with a mean knowledge score of 93.3% at 4 weeks (95% CI [83.3, 100.0]), with an absolute improvement of 28.7% from pre-intervention and a 4.6% decline from immediately post-intervention (Figure 3).

## 4. Discussion

The VR-based intervention explored in this study appeared both feasible and effective, indicating that VR-based interventions for patient education and procedural informed consent hold considerable promise. Fostering full study comprehension in patients via traditional written and verbal consent mechanisms has proven to be a challenging task for clinicians, as there is typically a literacy gap, language barrier, or anxiety that may affect patients’ comprehension ability. Patients may have difficulty making thoughtful decisions to participate in a study when comprehension is limited, and clinicians may find it difficult to determine how to best ensure that patients are appropriately informed of potential post-treatment complications [4,7].

### 4.1. Interpretation of Findings

Patients’ immediate improvement in understanding of the IV port placement procedure with the use of VR-based education of 33.3% and long-term improvement of 28.7% strongly suggest that virtual education is effective. Although the long-term knowledge improvement was not found to be statistically significant in this study, this may reflect the limitation of utilizing a three-question, open-ended questionnaire to measure knowledge; future assessments would benefit from using a more objective and detailed questionnaire. Additionally, it is unclear whether this technology improves procedure knowledge over traditional consenting mechanisms, as no comparisons were made to traditional consenting in this study. The literature reports that informed consent comprehension is generally low (<50%) [28]; however, the nature of the knowledge assessment, such as how many questions are asked and what topics are covered, likely has a significant effect on knowledge scores. As such, it is difficult to determine how VR-based consent compares to other mechanisms without a direct comparison study with appropriately implemented controls. The participants reported tolerating VR technology in this study without dizziness, disorientation, or vertigo, suggesting that patients tolerate and accept this form of digital education. This, combined with clinical staff operating VR with minimal expertise, promises that effective VR-based education and informed consent can be easily deployed, well accepted, and unburden physicians and nurses from performing these tasks. Another advantage of using VR for patient education and informed consent is that it can solve the issues of doctors’ inconsistency in language and excessive use of medical jargon, both of which contribute to patient confusion. The conversational format of VR can address the literacy gap that often exists with written informed consent forms and language barriers, as the software can readily switch between languages. Furthermore, eye-tracking capabilities in the VR environment can monitor what data the patient has viewed and offer additional information exposure if a critical piece of information has been overlooked (Figure 1C; Supplemental Video S2).

### 4.2. Generative Artificial Intelligence (AI) Technology

**Figure 4:**
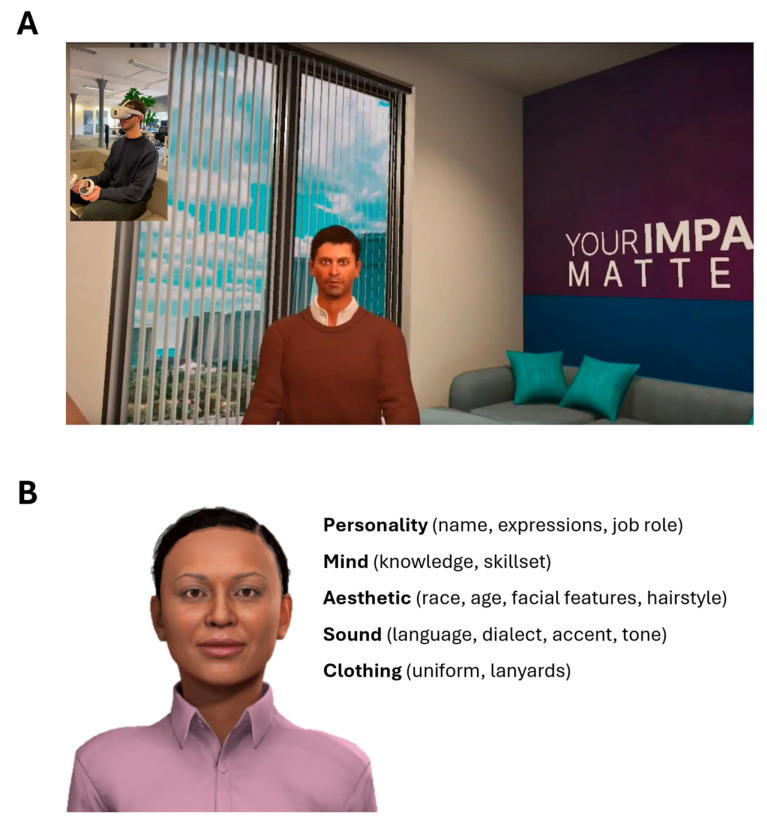
(**A**) Example of user interaction with AI-based VR technology for clinical trial consent; (**B**) example of available avatar customization options from Recourse AI for adaptive, AI-based VR technology.

While the technology in this study utilized a scripted format, a later embodiment using a generative artificial intelligence (AI) engine with immersive VR was also investigated (Figure 4A; Supplemental Video S3). These tools allow patients to engage in natural, two-way, voice-based conversations in real time with digital human-like avatars to gain personalized information on demand in a four-layer system. The first application experience layer allows users to interact with the avatar and environment via voice input, speaking naturally in their normal tone and with support for a wide variety of dialects and languages, and closed caption functionality is deployed for additional usability within the VR experience. The VR environment can be customized by content authors, and patients can toggle between different environments to select a space in which they feel the most comfortable. The AI backend architecture delivers faster response times and delivers higher performance at low latency than most generic chatbots do, such as those designed for customer service use cases, as these models are specifically designed for high-performance at low latency to maintain rapport and engagement with patients. The second layer of the digital human character is customizable and scalable so that this layer can align the avatar’s physical attributes with their “brain” and personality, which are optimized for realism. The digital character creator suite enables the development of entirely custom avatars from any demographic or gender and can even be based on a real human persona by leveraging digital twin AI cloning technology (Figure 4B). In addition, facial expressions and voices, including pitch and tone, are all configurable for further alignment, with advanced text-to-speech technology being leveraged. The ability to portray different visual personas with varying personalities and backgrounds is thought to facilitate a stronger emotional connection between the virtual agent and patient-user. Cost savings are also achieved here by removing the need for studio-based custom avatar generation, catering to many different needs without scaling costs. The third conversational AI “brain” layer is where the system’s interaction capabilities go beyond off-the-shelf chatbot solutions and scripted AI video generators, as it is optimized for real-time two-way conversation with configurable knowledge, resulting in a highly engaging, fully compliant experience. Specifically, the digital VR expert leverages only preapproved knowledge from its ‘brain’ (“research and community informed content”), with guardrails to avoid hallucinations (the “trust layer”), ensuring that patients receive correct information only from a trusted source. Each response can be referenced for “explainability” purposes, with users also able to provide real-time feedback on avatar responses. The system can also control the extent to which the digital expert responds to questions versus when a discussion with a human is offered for more sensitive or advanced queries. If a question is not strictly relevant to the conversation (e.g., small talk), the answer can reveal some personality and (if appropriate) even some humor. The fourth and final system layer provides a secure and compliant digital architecture that is optimized for the specific healthcare education use case, with the system having a rigorous data and information security framework, as all data are stored on reliable, secured, scalable databases, with client data only being used to train customer-specific AI models. For future studies of VR-based informed consent, the application of AI should be explored to provide the best possible user experience.

### 4.3. Ethical Considerations

Participants should be adequately informed about the use of VR prior to using it for receiving informed consent for a clinical trial, including the potential risks and benefits of VR use, and participants should consent to use of the technology. Alternative informed consent mechanisms should be made available for participants who decide to forego the use of VR. Although motion sickness was not reported among our study participants, this is a known risk of VR and should be discussed with participants prior to use [24]. Data obtained within VR, such as any audio/visual recordings of a participant, eye tracking, or other collected biometrics, should be treated as sensitive patient health information and managed accordingly. Finally, although the presented study here used a scripted format that was not generative, the use of generative AI for informed consent offers unique ethical challenges. Potential social biases stemming from how the generative AI model was trained [29] and data hallucination, that is the generative AI making incorrect or misleading statements, could negatively impact the use of such technology for informed consent, and significant validation efforts should be employed before the use of generative AI in a clinical setting.

### 4.4. Study Limitations

A limitation of this initial feasibility study is the small sample size, and further study is warranted. If a larger sample size yields similar feasibility results and statistical improvement in knowledge, then it would be reasonable to investigate the possibility of employing this technology in hospitals and clinics around the world where VR-based education is cost-effective. Notably, while this study targeted a variety of oncology patients, it did not include any patients with other diagnoses and informed them about only one clinical procedure. The electronic health information literacy of the evaluated patient population was moderate-to-high (Figure 2), and it is unknown whether a population with lower eHEALS scores would have similar knowledge retention scores. Furthermore, only English-speaking patients were recruited, which may have impacted the study results. Although minimal differences in health data literacy have been identified between English-speaking and limited-English proficiency patients, there may be differences in confidence in the use of technology to answer health questions, as one study revealed that 68% of English speakers “agreed/strongly agreed” that they knew how to use the internet to answer their health questions, whereas only 47% of Spanish-speaking participants agreed [30]. Larger-scale future studies should broaden the patient demographics, diagnoses, and interventions for which education is provided. Finally, this study did not measure pre-intervention anxiety, which may cause patients to not think clearly about what they are consenting to [4]. We cannot conclude whether anxiety was impacted by this technology in our study, but future studies would benefit from including anxiety as a metric. Encouragingly, the age range of patients was 50–88 years, indicating that older patients will be accepting of and effectively educated by this new and likely unfamiliar technology. However, this study does not show whether younger patients would yield similar improvements in comprehension. Other studies have included predominantly older patients (mean age 69 ± 8 years) who had no prior experience using VR and who reported that VR is a useful tool in healthcare and helped them to feel more informed about an upcoming procedure, supporting the results of this study [31]. Notably, the patient population of the current study does not allow us to predict acceptance or effectiveness among sub-populations such as pediatric patients. Finally, this study did not evaluate the psychological impact of the VR experience on participants and how receiving informed consent through this mechanism may impact consent decisions. Future studies would benefit from a psychological component to understand how this technology impacts decision-making and emotional responses to clinical trial participation.

### 4.5. Implications for Practice

Overall, VR can solve the issues of communicating complex medical information to patients while bridging the literacy gap that often exists with written informed consent forms and addressing possible language barriers, as software settings can be easily programmed to change languages when necessary. Oncology clinical staff reported the VR technology in this study was easy to use with minimal expertise required, offering encouraging results that this technology could be used in clinical practice. As this study was a feasibility assessment for general technology use, and not necessarily operational implementation, an important consideration for clinical use is the cost of VR technology and adoption in the clinical environment will likely require detailed cost–benefit analyses.

### 4.6. Future Research Considerations

Regarding technological challenges, some participants reported the audio volume was low and sometimes difficult to hear in our study. Auditory and visual acuity were not accounted for in our study population, which may be a factor to consider for future studies assessing VR informed consent. Further, when devising comparison studies against traditional consenting mechanisms, researchers should consider a calibration step for participants prior to administering informed consent to ensure all audio, visual, and tactile input are adequate to account for potential sensory impairments.

A large contributor to receiving subpar informed consent from patients is pre-intervention anxiety, which may cause patients to not think clearly about what they are consenting to, and unfortunately, this feasibility study did not produce any data on whether anxiety levels were impacted. Future follow-up studies should include this as a metric to determine how VR affects the mental clarity of patients who use digital technology platforms. Additionally, this feasibility study focused primarily on the feasibility of implementing this technology in a clinical setting and assessing patient tolerability and did not make any comparisons to traditional written and verbal consent mechanisms. With this technology demonstrating high tolerability and easy clinical deployment in this study, future studies should look to compare knowledge acceptance and retention using VR-assisted informed consent versus traditional consenting mechanisms to determine if this technology outperforms conventional methods. It is reasonable to conclude that if future studies show that people with varying income levels, cultural backgrounds, ethnicities, and different types of illness and procedures show similar improvements in comprehension, then hospitals around the world should consider the cost and practicality of employing VR to receive informed consent from patients in their clinics.

The assessment of alternative digital technologies, such as phone or web applications, using a similar conversational educational framework would be valuable in cases where VR is inaccessible or cost-prohibitive. While VR has the benefit of providing an immersive experience, which studies have shown improves knowledge retention [32], alternative delivery methods could still offer significant value with a lower barrier of entry and should be explored in future studies.

## 5. Conclusions

In this feasibility study, the assessment of 16 oncology patients revealed that VR-assisted informed consent for an IV port placement procedure was feasible, well tolerated, and proven to be a useful tool for improving patients’ comprehension of the procedure. Furthermore, the results of this study support that VR technology can be effectively deployed in a clinical oncological setting. Use of VR technology provides numerous benefits to both patients and clinicians for the implementation of the informed consent process, including the ability to bridge literacy gaps and language barriers. Limitations of this study include the small sample size, single clinical environment, older participant population, and lack of comparison to traditional consenting mechanisms. Larger-scale studies are warranted to further evaluate effectiveness for patients, compare VR with traditional consent mechanisms, and determine generalizability to diagnoses outside of oncology and to broader patient demographics.

## Data Availability

Dataset available on request from the authors.

## Supplementary Materials

The following supporting information can be downloaded at the following link: https://www.mdpi.com/article/10.3390/cancers17071148/s1, Video S1: Volumetric computation of VR avatar; Video S2: Informed consent virtual reality experience example; Video S3: Interactive AI experience example.
